# Ununited Fractures

**Published:** 1857-12

**Authors:** Charles Brackett

**Affiliations:** Rochester, Fulton Co., Indiana


ARTICLE II.

### UNUNITED FRACTURES.

BY CHARLES BRACKETT, M.D., OF ROCHESTER, FULTON CO., INDIANA.

November 12th, 1857.

Sometime during early summer, George Lawson, of Marshall County, came to me with an ununited fracture of the left thigh. I explained to him the different modes of relieving such difficulties, and told him that during that or the first of the next week I would operate. He promised to come down the next week. Three weeks afterward he sent to me from Plymouth to see him and amputate the limb. At the request of the surgeon attending, I took my amputating case, went up, and found that he was under the care of a homœopath, who had performed the following operation on the thigh two weeks previously: First, after trying unsuccessfully to produce anæsthesia, he made a free incision of about six inches in length over the seat of fracture, on the anterior face of the limb, at the junction of the lower with the middle third; then, with a straight saw, chisel and forceps, he succeeded in removing a piece the lower end of the superior fragment; then, as the fingers of assistants and the saw were too large to reach the upper portion of the lower fragment, which was posterior, he proposed to turn the patient over and make another incision on the posterior face of the thigh, that the bone might be more easily reached with the saw. As, however, the day was nearly passed, and assistants and patient pretty thoroughly fatigued, the operation was concluded by extending the limb, placing the ends in apposition, on a long board reaching from the hip to the foot, with a foot board attached. When I saw him, matter was discharging freely from the thigh; the bones lapped several inches more or less. Having much faith in the *vis medicatrix naturæ*, I applied the bandage *amidonne* the whole length of the limb, directing that the limb should, for at least ten weeks longer, be kept immovably fixed by immovable bandage, and that at the expiration of three weeks I should be sent for, when I would apply one of Day's carved splints, which would allow the limb to be partially flexed, and with the starched bandage enable Lawson to go about with crutches, with no danger of further displacement.

I gave him some encouragement that the limb might become serviceable, and condemned at any rate the removal of the leg at that time. I heard nothing further from him for seven weeks, when he again sent for me to see him at his own residence. He was still in bed, as he had been since I had seen him, with the limb tightly bandaged from the knee up four or five inches above the fracture with four short splints and a common bandage. The thigh was so tightly bandaged, that it was a matter of wonder to me that the leg did not die from the effects of it; but, instead, the leg and foot were not the least swollen, and only a slight redness on the inner side of the knee. I refused to remove the bandages until at the expiration of three weeks more, giving him my reasons for thus doing. At the end of another week he sent for me again to remove the leg. I sent my partner, Dr. Gould, with instructions to direct him to wait till the expiration of the time I had fixed. Lawson consented; and when the time was up, I went out and removed the dressings, especially, as from the pressure of the inner splint, an abcess was forming at the seat of the redness above mentioned. We could not tell to a certainty whether the union was sufficiently firm to bear his weight, as the muscles and other tissues were so wasted from long pressure and disuse that he could not try it. To *appearance*, however, the limb is firm at the point of fracture. I opened the abcess some two weeks later, in Rochester, whither he had come to see me, or rather it opened itself about the time I got to the house. It is a matter of uncertainty yet whether the bone has become diseased, as it has been discharging considerably from the time I saw him at Plymouth to the present.

There are several points of interest connected with this case, not the least remarkable of which is that any man could dare undertake an operation of the kind unprovided with chain saws, retractors and other appliances, which should render the *operation* perfect, even if a cure should not follow. If a cure or recovery takes place in the leg, it is a marked instance of what nature can do under difficulties.

You shall have the sequel to this case, if we live to observe it.

Mrs. Putman, eight miles east of town, was confined under the care of Dr. S. S. Terry, and delivered of twins. One week after, Dr. Robbins, of Rochester, was called in consultation, and by him, the next day, I was called to see as to the propriety of amputation—gangrenous erysipelas having attacked the left leg and the skin sphacelated to the knee, with most copious and exceedingly offensive discharge; pulse about one hundred and thirty, small and wiry; spots of erysipelas showing themselves on right leg, one arm, and on the upper portion of sternum. We waited one day to see if *large doses of quinine and opium*

internally, and iron or iodine tinct. sat. locally would check the spread of the disease. It appeared to do so, and the next day we proceeded to amputate about six inches above the knee. I chose the circular method, and cut just above where a spot of disease showed itself on the thigh; when down to the periosteum and through it, it slipped up the bone like rotten epidermis. I thought "my patient will surely die," but finished the operation, and at the end of ten days she was quite smart. All the other spots of inflammation had suppurated freely, had not spread, and the patient every way comfortable, able to sit up, with good appetite and spirits. The stump healed by first intention. The same disease attacked her husband, and was with little difficulty controled by *iron, qui. sul. and opium* internally, with tinct. iod. sat. or sol. ferri. sul. locally applied, both being used, though I placed most confidence in sul. ferri. In both these cases *quinine* exerted a marked *beneficial influence.* It was omitted several times during the treatment, *always* followed by an aggravation of the symptoms, which immediately became ameliorated on a recurrence to the quinine. The skin (on the leg removed) from metatarsus to knee was separated from the superficial fascia and entirely black except a small strip on posterior surface. I exposed it to a stream of running water for eighteen hours to cleanse it. All through the black sphacelated skin, the vessels were seen entirely unharmed by disease, and were the only connecting links between the skin and deep-seated tissues. The disease had reached the bone at several points on the tibia, evidenced by discoloration and softening.

*May 26th*, 1857.—Mary Tully, aged 14, came to me, quite blind. Scrofulous diathesis; lids so puffed that I could not see eyes. Eyes were always weak; intolerant of light; acutely inflamed two months. Apply nit. argt. to brow, sweet oil to edges of lids; cathartic of elaterin. Three days after can see eye-balls; cornea quite opaque; whole conjunctiva red, and dotted with minute spots of ulceration. Use aqueous sol. sul. morph. aud sul. qui. to eyes four times per day. Continue oil to edges of lids, cathartics of elaterin, cret. preparat. and prot. iod. hydg. and aloes alternately. Continue treatment five weeks. Discharged cured, except tumor on right eye-ball at lower edge of cornea, of size of a small pea. Continue wash as above, and same cathartics once a-week. *Sept.* 4*th.*—Returned to see me, vision good, eyes strong; tumor, which was developed by internal inflammation, entirely gone, and general health good.

